# Long-Term Exercise Protects against Cellular Stresses in Aged Mice

**DOI:** 10.1155/2018/2894247

**Published:** 2018-03-25

**Authors:** Irina Belaya, Masataka Suwa, Tao Chen, Rashid Giniatullin, Katja M. Kanninen, Mustafa Atalay, Shuzo Kumagai

**Affiliations:** ^1^Institute of Biomedicine, University of Eastern Finland, Yliopistonranta 1 E, 70211 Kuopio, Finland; ^2^Faculty of Life Design, Tohoku Institute of Technology, 6 Futatsusawa, Taihaku-ku, Sendai, Miyagi 982-8588, Japan; ^3^Health Support Center WELPO, Toyota Motor Corporation, 1-1 Ipponmatsu, Iwakura-cho, Toyota, Aichi 444-2225, Japan; ^4^Faculty of Arts and Science and Graduate School of Human-Environment Studies, Kyushu University, Kasuga, Fukuoka, Japan; ^5^A. I. Virtanen Institute for Molecular Sciences, University of Eastern Finland, Neulaniementie 2, 70211 Kuopio, Finland

## Abstract

The current study examined the effect of aging and long-term wheel-running on the expression of heat shock protein (HSP), redox regulation, and endoplasmic reticulum (ER) stress markers in tibialis anterior (T.A.) and soleus muscle of mice. Male mice were divided into young (Y, 3-month-old), old-sedentary (OS, 24-month-old), and old-exercise (OE, 24-month-old) groups. The OE group started voluntary wheel-running at 3 months and continued until 24 months of age. Aging was associated with a higher thioredoxin-interacting protein (TxNiP) level, lower thioredoxin-1 (TRX-1) to TxNiP ratio—a determinant of redox regulation and increased CHOP, an indicator of ER stress-related apoptosis signaling in both muscles. Notably, GRP78, a key indicator of ER stress, was selectively elevated in T.A. Long-term exercise decreased TxNiP in T.A. and soleus muscles and increased the TRX-1/TxNiP ratio in soleus muscle of aged mice. Inducible HSP70 and constituent HSC70 were upregulated, whereas CHOP was reduced after exercise in soleus muscle. Thus, our data demonstrated that aging induced oxidative stress and activated ER stress-related apoptosis signaling in skeletal muscle, whereas long-term wheel-running improved redox regulation, ER stress adaptation and attenuated ER stress-related apoptosis signaling. These findings suggest that life-long exercise can protect against age-related cellular stress.

## 1. Introduction

Ageing is associated with the accumulation of anatomical and molecular changes that promote muscle atrophy, which is associated with a number of chronic diseases [1]. One of the most important steps in the prevention of age-related diseases and in the promotion of healthy aging is to increase knowledge of the molecular mechanisms associated with aging; it is also crucial to reveal how a healthy lifestyle, including regular physical exercise, may improve these processes. Cellular senescence is associated with impaired calcium homeostasis, mitochondrial dysfunction, aberrant redox control of cellular signaling, elevated oxidative and endoplasmic reticulum (ER) stress, a dysregulated unfolded protein response (UPR), and altered protein homeostasis [2]. Although the production of reactive oxygen species (ROS) is essential in physiological homeostasis and optimal muscle contraction, in times of oxidative stress, the increased ROS production can disrupt redox regulation of protein turnover, leading to increased protein misfolding and aggregation [3, 4]. While sustained deviation from redox homeostasis and activation of ER stress have been claimed to promote the development of aged-related diseases [3, 5], there is limited direct evidence to support this hypothesis.

The endogenous thiols including the glutathione (GSH) and thioredoxin (TRX) systems together with thioredoxin-interacting protein (TxNiP), an endogenous inhibitor of TRX, are critical components of redox signaling and in the regulation of protection against oxidative stress [6]; they have also been associated with the regulation of UPR [7]. It has been shown that the GSH concentration is decreased during aging in skeletal muscle [8–10]. To date, there is limited information available regarding the effect of aging on the TRX system; in the only publication on this issue, the TRX protein content was higher in skeletal muscle of old mice compared with their younger counterparts [11]. To the best of our knowledge, age-dependent changes in the TxNiP system in skeletal muscle have not been investigated.

In the ER, lumen protein folding and homeostasis are carried out by chaperones and oxidatively folding enzymes including glucose-regulated protein 78 (GRP78, also known as BiP), and 94 (GRP94), calnexin, and thiol-disulfide oxidoreductase—protein disulphide isomerase (PDI), which belongs to the TRX superfamily [3]. During aging, the functional efficiency of ER chaperones declines, resulting in an accumulation of unfolded or misfolded proteins [12]. This process induces ER stress and leads to an activation of the UPR. The UPR system resolves ER stress by activating several signaling pathways aiming to restore protein homoeostasis by (1) increasing the synthesis of protein chaperones, (2) enhancing protein folding capacity, (3) stimulating protein degradation, and (4) decreasing protein production [3]. Additionally, cytoplasmic and mitochondrial chaperones, the heat shock proteins (HSPs), play a critical role in protein folding, intracellular trafficking of proteins, as well as dealing with proteins denatured by heat and metabolic stresses [13, 14]. It is known that aging suppresses HSP responses in skeletal muscle [15]. Chung and Ng [16] have detected increased HSP basal levels in the skeletal muscle of old rats, which can be linked to increased oxidative stress in sedentary muscle [17]. Several studies have reported that HSPs play an important role in apoptotic regulation. It has been demonstrated that a reduction in the level of HSP70 leads to ER and sarcoplasmic reticulum (SR) stress signaling and apoptosis induction in the skeletal muscle of aged mice [18]. The mitochondrial protein, HSP60, is also involved in antiapoptotic regulation [19]. Therefore, changes in HSP expression may influence apoptosis in skeletal muscle during aging.

Several age-related diseases are associated with chronic ER stress or impairment in UPR and HSP responses; these are reflected in overexpression of the transcription factor CCAAT-enhancer-binding protein homologous protein- (CHOP-) mediated, ER-originated proapoptotic signaling pathways [3]. Numerous studies have described induction of ER stress and ER stress signaling apoptosis pathways during aging. Levels of GRP78, PDI, and CHOP are increased in skeletal muscle tissues of old animals as compared to younger animals [16, 18]. On the other hand, O'Leary et al. [20] detected a 17-fold increase in CHOP protein expression, whereas a marginal decrease in GRP78 protein expression was observed in extensor digitorum longus (EDL) muscle of aged mice compared to younger animals. In addition, there are other studies reporting a reduction in the GRP78 level and an increase in the CHOP level during aging in various tissues including the brain [21], liver, kidney, heart, and spleen [22]. The rather sketchy information in the literature regarding ER stress and ER stress-related apoptosis in skeletal muscle during aging underlines the need for further studies in order to achieve a more thorough understanding of the topic.

Regular exercise improves the physical capacity and reduces the risk of developing chronic and age-related diseases [21, 22] by improving the metabolic state, antioxidant protection, and redox regulation [23]. Tarpenning et al. [24] examined the changes in the skeletal muscle of master athletes, who exercised regularly for 20 years. Lifelong training was reported to slow down aging-associated skeletal muscle fiber atrophy and prevent the reduction in muscular strength. Notably, acute intensive exercise induces the production of ROS that can evoke macromolecular damage, oxidative stress, ER stress, and activation of the UPR [6]. On the other hand, regular exercise training results in adaptations in antioxidant defense and improves redox signaling [6] to protect cells against stress-related diseases, thus delaying the aging processes [25]. In addition, the UPR, which is activated by exercise in skeletal muscles, may exert protective effects against ER stress and can promote metabolic adaptation to physical activity [26]. Long-term exercise was reported to upregulate HSP production in skeletal muscle [15, 25], which would be beneficial in coping with oxidative stress, ER stress, and ER stress-related apoptosis. Nevertheless, the ability to induce HSPs in aged skeletal muscle is compromised, which may impair the exercise-mediated adaptation processes [27].

There is only limited information available on the association of aging and exercise training concerning oxidative stress, ER (SR) stress, UPR, and/or ER stress-related apoptosis in skeletal muscle. The question of whether exercise training can reduce metabolic stress and apoptosis in skeletal muscle by increasing chaperone expressions and improving redox regulation has not been answered adequately. Our hypothesis is based on the fact that there is an age-induced disruption of redox regulation, increased redox ER stress, and ER stress-related apoptosis, and that long-term exercise can exert protective effects against these processes. The novelty of our study is that we investigated the key molecular markers associated with redox state, ER stress, and apoptosis in skeletal muscle of old animals in a life-long running model and compared them to young animals. Moreover, we determined age-related and exercise-induced changes in two types of skeletal muscle tissue: soleus, mostly composed of slow-twitch, aerobic muscle fibers, and tibialis anterior (T.A.), mostly consisting of fast-twitch glycolytic muscle fibers in order to reveal possible fiber specific changes.

## 2. Materials and Methods

### 2.1. Animals and Design

Three-month-old male ICR mice (CLEA Japan Inc., Tokyo, Japan) were maintained under standard conditions (24 ± 1°C; 12 h light–dark cycle) and had free access to food (CLEA Japan, CE-2, 3449 kcal/kg) and water. Male mice were used in order to avoid postmenopausal effects such as severe changes of sex hormones and osteoporosis in this life-long study. All mice were divided into three groups: young (Y, *n* = 12), old sedentary (OS, *n* = 5), and old exercise (OE, *n* = 5). Mice from the Y group were sacrificed at 3 months age to obtain T.A. and soleus muscle samples. The mice in the OS and OE groups were maintained in individual wire mesh cages (90 × 220 × 90 mm). The mice from the OE group had free access to an activity wheel (628 mm circumference, 50 mm wide running surface of wire mesh; Shinano Instruments, Tokyo, Japan) from 3 to 24 months of age. Muscle samples from the mice in OS and OE group were collected when the mice were 24-month-old.

The mice were sacrificed with an overdose of pentobarbital sodium (60 mg/kg body weight intraperitoneally). The mice from the OE group were sacrificed 2 days after the last wheel-running session to rule out any effect of acute exercise. The T.A. and soleus muscles were removed, weighed, immediately frozen in liquid nitrogen, and stored at −80°C for later homogenization and biochemical assays. The experimental protocol was approved by the University Animal Experiment Committee and conducted in accordance with the Tohoku Institute of Technology guidelines for the Care and Use of Laboratory Animals.

### 2.2. Sample Preparation and Western Blot Analysis

Muscle samples were homogenized in eight volumes of lysis buffer (pH 7.4) containing 10 mM NaCl, 1.5 mM MgCl_2_, 20 mM HEPES, 20% glycerol, 0.1% Triton X-100, 5% protease inhibitor cocktail, 0.5% PMSF, and 1.5% phosphatase inhibitor cocktail 2 (Sigma-Aldrich, St. Louis, MO, USA). Cytosolic extracts from samples were centrifuged at 2500 rpm for 10 min at +4°C. Supernatants containing cytosolic proteins were aliquoted prior to further measurements. Protein concentrations were measured by using the BCA protein assay kit (Thermo Fisher, Rockford, IL, USA). Western blot procedures were as previously reported [13], proteins were separated by SDS-PAGE, transferred to a nitrocellulose membrane (Millipore, Bedford, Mass., USA), and incubated for 1 hour at +37°C in blocking buffer (100 mM Tris, 154 mM NaCl, 5% nonfat dry milk). After treating with the antibodies listed below overnight at +4°C, the membranes were washed 4 times per 5 min with Tris-buffered saline containing 0.1% Tween 20 and incubated in secondary antibodies (antimouse IgG, 35521; anti-rabbit IgG, 35571; anti-rat IgG, A-21096, Thermo Fisher, Rockford, IL, USA) for 30 min in room temperature. GAPDH (sc-25778) was used as internal standard. Proteins were visualized with the Odyssey Imaging System (LI-COR Biosciences Inc., Lincoln, NB, USA) and quantified using Odyssey software.

### 2.3. Primary Antibodies

Antibodies against heat shock protein 25 (HSP25, SPA-801), heat shock protein 60 (HSP60, SPA-806), heat shock protein 70 (HSP70, SPA-810), heat shock protein 90 (HSP90, SPA-835), glucose-regulated protein 78 (GRP78, SPA-826), and glucose-regulated protein 75 (GRP75, SPA-825) were purchased from Enzo Life Sciences Inc., (Farmingdale, NY, USA). The antibody against cytosolic thioredoxin-1 (TRX-1, ATRX-06) was purchased from IMCO Corp (Stockholm, Sweden), thioredoxin-interacting protein (TxNiP, K0205-3) from MBL (Medical and Biological Laboratories Co. Ltd, Nagoya, Japan), and 4-hydroxy-2-nonenal (4-HNE, HNE11-S) from Alpha Diagnostic IntI Inc. (San Antonio, TX, USA). Antibodies against CCAAT/enhancer-binding protein homologous protein (CHOP, L63F7) and protein disulfide isomerase (PDI, C81H6) were purchased from Cell Signaling Technology (Danvers, MA, USA). The antibody against GAPDH (sc-25778) was purchased from Santa Cruz Biotechnology, Inc. (Santa Cruz, CA, USA).

### 2.4. Protein Carbonyls

The level of protein carbonyls, a marker of protein oxidative damage, was measured from plasma samples after derivatization with dinitrophenylhydrazine using an ELISA method previously described with modifications [28]. The maximal intra-assay CV for protein carbonyls was 5.9% and the maximal inter-assay CV was 9.2%.

### 2.5. Lipid Hydroperoxide Assay

Lipid hydroperoxides (LPO) in muscle tissue were determined as described previously [29]. This method is based on oxidation of Fe II to Fe III by lipid hydroperoxides under acidic conditions, followed by complexation of Fe III by xylenol orange.

### 2.6. Statistical Analyses

All data are expressed as the mean ± SEM. The statistical significance of the data was determined using one-way analysis of variance (ANOVA) with a posthoc test (LSD). The equality of variances was analyzed with Levene's test. The nonparametric Mann–Whitney test was applied for nonhomogenously distributed data. Spearman's test was used for correlation analysis. Statistical analyses were performed with SPSS software version 21.0, and the level of significance was set at *p* < 0.05.

## 3. Results

No significant differences in body mass were observed among the groups [30]. The total food intake was 15% higher in OE group compared to the old-sedentary OS group [30]. The T.A. mass and T.A. mass/body mass were significantly lower in old mice, while wheel-running partly restored age-related decreases in T.A. mass (unpublished data). Notably, soleus mass and soleus mass/body mass were significantly lower in the OS group than in the Y group. Furthermore, those in the OE group were significantly higher than in the OS group, thereby indicating the efficiency of wheel-running [30].

### 3.1. Effects of Aging on Redox Regulation, HSP Expression, ER Stress, and UPR in Skeletal Muscle

**Figure 1:**
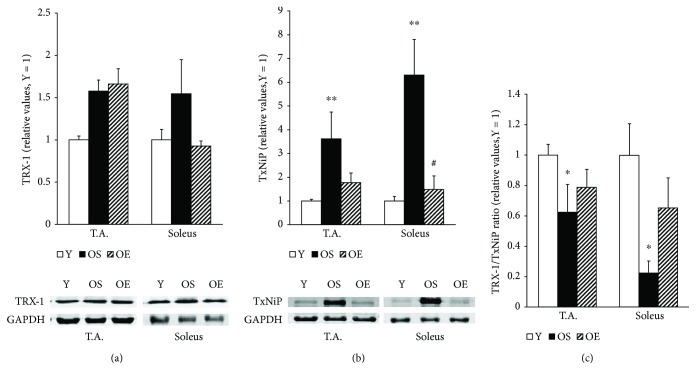
Effect of aging and voluntary wheel-running on the following redox regulation markers; thioredoxin-1 (TRX-1), thioredoxin-interacting protein (TxNiP), and TRX-1/TxNiP ratio in T.A. and soleus muscles of mice. White bars: young 3-month-old mice; black bars: OS sedentary 24-month-old mice; diagonal-striped bars: OE 24-month-old mice after 21 months' voluntary wheel-running. Data are mean ± SEM. *n* = 12 for Y, *n* = 5 for OS, and *n* = 5 for OE. Difference due to age: ^∗^*p* < 0.05, ^∗∗^*p* < 0.01. Difference due to exercise: ^#^*p* < 0.05.

Ageing remarkably increased TxNiP protein expression by 261.8% in the fast-twitch glycolytic T.A. muscle fibers and by 530.2% in the slow-twitch, aerobic soleus muscle fibers over the level in Y mice (*p* < 0.01, Figure 1). In OS mice, the level of TRX-1 expression was slightly increased by 57.7% in T.A. muscle (*p* > 0.05) and by 54.5% (*p* > 0.05) in soleus muscle compared to Y mice (Figure 1). Notably, we detected a decrease in the TRX-1/TxNiP ratio by 37.6% in T.A. muscle (*p* < 0.05) and by 77.6% in soleus muscle (*p* < 0.05) in OS mice compared to Y mice (Figure 1). We observed no significant changes in the level of protein carbonyls, 4-HNE adduct (lipid peroxidation marker), and lipid hydroperoxides (LPO) (Table 1) in skeletal muscle of old mice as compared to the young mice.

**Figure 2:**
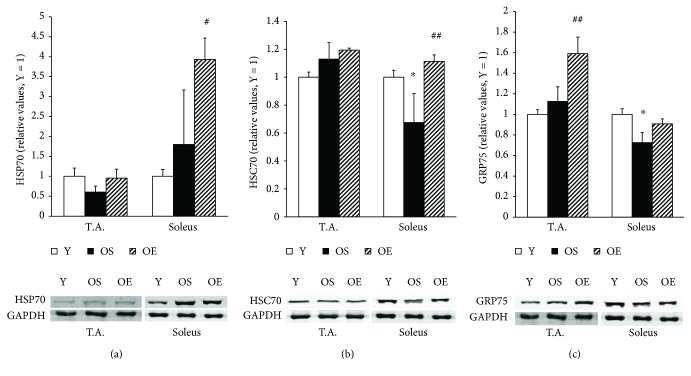
Effect of aging and voluntary wheel-running on the expressions of HSP70, HSC70, and GRP75 heat shock proteins in T.A. and soleus muscles of mice. Groups and bars are as in Figure 1. Data are mean ± SEM. *n* = 12 for Y, *n* = 5 for OS, and *n* = 5 for OE. Difference due to age: ^∗^*p* < 0.05. Difference due to exercise: ^#^*p* < 0.05, ^##^*p* < 0.01.

Moderate reductions were observed in the GRP75 (27.5%, *p* < 0.05) and HSC70 (32.5%, *p* < 0.05) expression levels in soleus muscle of OS mice as compared to the Y mice (Figure 2). However, aging did not affect the expression levels of HSP25, HSP60, and HSP70 proteins in T.A. or soleus skeletal muscles (Table 1, Figure 2).

**Figure 3:**
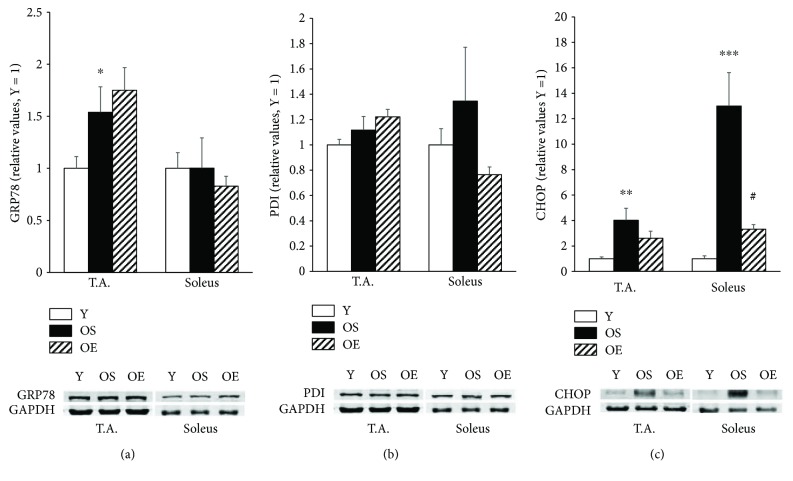
Effect of aging and voluntary wheel-running on ER stress and UPR markers GRP78, PDI, and CHOP in T.A. and soleus muscles of mice. Groups and bars are as in Figure 1. Data are mean ± SEM. *n* = 12 for Y, *n* = 5 for OS, and *n* = 5 for OE. Difference due to age: ^∗^*p* < 0.05, ^∗∗^*p* < 0.01, ^∗∗∗^*p* ≤ 0.001. Difference due to exercise: ^#^*p* < 0.05.

To understand the cellular stress state of aged mice in view of ER stress and UPR, we tested the levels of GRP78 chaperone, PDI, and CHOP proteins (Figure 3). In T.A. muscle of OS mice, the level of GRP78 expression was increased by 53.9% (*p* < 0.05) compared to Y mice, whereas the GRP78 level was not changed in soleus muscle. PDI protein levels remained unaltered in OS mice in both T.A. and soleus muscles compared to Y mice. In OS mice, the level of the ER stress-related apoptosis marker CHOP was significantly elevated in T.A. muscle (*p* < 0.01) by 302.1% and in soleus muscle (*p* ≤ 0.001) by 1199.8% compared to Y mice.

### 3.2. Effects of Long-Term Exercise on Redox Regulation and HSP Expression in Skeletal Muscle of Old Mice

Long-term exercise decreased the TxNiP level in both muscle types in the aged mice (Figure 1)—in T.A. by 50.9% (*p* > 0.05) and in soleus muscle by 76.4% (*p* < 0.05). The TRX-1/TxNiP ratio was increased by 26.3% in T.A. (*p* > 0.05) and by 190.4% in soleus muscle (*p* > 0.05) of OE mice compared to OS mice (Figure 1). Moreover, after long-term exercise, the level of the 4-HNE adduct (lipid peroxidation marker) declined by 39.3% (*p* < 0.05) compared to OS control mice in T.A. muscle (Table 1). Our findings did not reveal any significant effects of long-term exercise on the LPO and amounts of protein carbonyls, a marker of oxidative damage in proteins, in skeletal muscle. (Table 1). In addition, no significant changes in the TRX-1 protein level were observed in both T.A. and soleus muscles after long-term exercise in OE mice as compared to OS mice (Figure 1).

We next examined mitochondrial HSP protein expressions—HSP60 protein and GRP75 chaperone. When compared to OS muscles, the exercise-trained T.A. muscle of old mice showed a marginal increase (21.6%, *p* > 0.05) in HSP60 protein expression and a more significant increase (41.1%, *p* < 0.01) in GRP75 protein expression (Table 1, Figure 2). The levels of these two mitochondrial HSP proteins strongly correlated with each other in T.A. muscle of old mice (*r* = 0.867; *p* < 0.01). At the same time, we did not observe any changes in these markers in soleus muscle of trained mice.

Several changes were observed in stress-inducible HSP70, HSP25, and housekeeping HSC70 proteins in soleus muscle of OE mice. After exercise, the level of HSP25 was slightly increased (43.1%, *p* > 0.05) in soleus muscle of old mice (Table 1). The expression of HSC70 was also significantly increased in soleus muscle of OE mice by 64.8% (*p* < 0.01) compared to OS mice (Figure 2). The expression of HSP70 in soleus muscle was also higher (118.5%, *p* < 0.05) in OE mice compared to OS mice (Figure 2). However, we did not observe any evidence that long-term exercise induced any significant changes in the expression levels of HSP25, HSC70, and HSP70 proteins in T.A.

### 3.3. Effects of Long-Term Exercise on ER Stress and UPR in Skeletal Muscle of Old Mice

To understand the effect of 21 months' wheel-running exercise on ER stress and UPR in skeletal muscle of old mice, we measured the levels of GRP78, PDI, and CHOP. Marginal upregulations of GRP78 (13.7%, *p* > 0.05) and PDI (9.4%, *p* > 0.05) proteins were observed to occur upon long-term exercise in T.A. muscle of OE mice (Figure 3). Furthermore, the levels of these two ER stress-related proteins remained unaltered in soleus muscle of OE mice compared to OS mice. Despite nonsignificant changes in GRP78 expression, the ER stress-related apoptotic marker CHOP was clearly attenuated by 74.5% (*p* < 0.05) in soleus muscle of trained old mice compared to their OS counterparts. The level of CHOP seemed to be lower (35.2%, *p* > 0.05) in T.A. muscle of the OE mice.

### 3.4. Correlation Analysis

We calculated the correlations among all studied cellular stress markers.  Table 2 lists the most significant correlations between redox regulation markers and ER stress markers.

## 4. Discussion

The present study describes a significant age-related suppression of antioxidant defense, impairment of redox regulation, an increase of ER stress and ER stress-related apoptosis, which are partly restored by long-term voluntary exercise.

The TRX system plays a crucial role in redox signaling and antioxidant defense. Under oxidative stress conditions, TRX exerts protective effects against apoptosis [6]; conversely, TxNiP inhibits the TRX system and promotes apoptosis. Thioredoxin-interacting protein directly binds to TRX and suppresses its reducing activity, and thus a lower TRX/TxNiP ratio may be a determinant of disrupted redox regulation and related disorders [31]. Furthermore, a dysfunction of thiol redox circuits, including TRX system, has been proposed to be the main cause of impaired redox homeostasis and dysregulation of cellular processes [6]. The present study is the first to determine changes in the TRX-TxNiP system in skeletal muscle of old mice. Notably, we observed a significant increase in the TxNiP protein content in both soleus and T.A. muscles of old animals. An important new finding was that aging significantly decreased the TRX-1/TxNiP ratio in both type of skeletal muscle tissues. The observed increases in the levels of TRX-1 (over 50%) in both skeletal muscle types in response to aging did not quite achieve statistical significance, possibly due to limited number of animals and the subsequent lack of statistical power. A previous study demonstrated a significant increase in the TRX-1 content in T.A. muscle of 28-month-old mice when compared with 6-month-old mice [11]. Despite the changes in TRX-1/TxNiP ratio and TxNIP and TRX-1 levels, no difference in protein carbonyl and 4-HNE protein adduct levels were observed between young and old sedentary animals. The lack of difference in oxidatively modified protein levels can be explained by their increased removal. The accumulation of oxidatively damaged proteins is also known to induce proteasome activity intended to achieve their removal [32]. Furthermore, we observed 21–26% increases of the lipid peroxidation marker LPO in old animals in T.A. muscle; however, these increases did not reach statistical significance. Nevertheless, our data collectively revealed increased TxNiP levels and a lower TRX-1/TxNiP ratio in aged animals that may explain the redox dysregulation and consequent cellular dysfunctions that occur during aging.

We evaluated the levels of ER stress and ER stress-specific apoptosis markers during aging and after voluntary exercise. The level of GRP78 significantly increased in T.A. but not in soleus muscle upon aging. GRP78 is a purely ER-located chaperone and its increased levels reflect an upstream stimulation of the unfolded protein response; unexpectedly, GRP78 levels increased only in T.A. muscle with aging. On the other hand, it has also been reported that during aging, the increased anabolic resistance and the downregulation of the protein folding capacity are more prominent in the soleus muscle compared to other types of skeletal muscle [33]. Nevertheless, a minor increase in the level of PDI—a key enzyme responsible for oxidative protein folding during ER stress and UPR—was observed in soleus and T.A. muscles upon aging. Furthermore, CHOP expression was remarkably increased in both soleus (13-fold) and T.A. (fourfold) muscles with aging. These findings of increased CHOP expression with aging are consistent with a previous study which demonstrated a significant upregulation of GRP78 and CHOP in the soleus muscle of 32-month-old rats as compared to 6-month-old rats [18]. Moreover, Hwee et al. [34] reported a significant increase in the expressions of GRP78, PDI, and CHOP in gastrocnemius muscle of 24-month-old mice in comparison with 6-month-old mice. Interestingly, a recent study indicated that CHOP levels were higher in both T.A. and soleus muscles of old mice, while the GRP78 expression demonstrated skeletal muscle type-dependent diverse changes during aging [33]. Therefore, regardless of the diversity, the results presented by others and us imply that there is an age-induced activation of UPR and ER stress-related apoptosis signaling in skeletal muscle.

In order to clarify the association of impaired chaperone function with increased ER (SR) stress in aging, we examined HSP levels in skeletal muscle as a function of age. The levels of mitochondrial chaperone GRP75 protein and constitutively expressed cytosolic HSC70 declined in soleus muscle upon aging. We did not detect any significant changes in the expression level of HSP25, HSP60, and HSP70 proteins in both T.A. and soleus muscles during aging. Various studies have reported somewhat conflicting age-related HSP expression changes in skeletal muscle. Valls et al. [35] stated that aging did not induce changes in HSP27 and HSP70 levels in skeletal muscle. In addition, there were no differences in the levels of HSP70 in skeletal muscles including the soleus, plantaris, and gastrocnemius muscles in aged rats [36]. Other studies have demonstrated conflicting results; a significant decrease in HSP70 expression without any change in the expression level of HSC70 in soleus muscle of aged rats [18] or even increased levels of HSP25, HSP60, and HSP70 in skeletal muscle of old rats compared to adult rats [16]. The discrepancies of these results may be due to differences in the age of animals and species used for the studies. Based on our results, we can only postulate that age-induced disturbances in redox regulation may impair HSP functions and reduce the level of HSPs.

The present study focused on the protective effect of long-term exercise in mice; our animals were trained for 21 months, the majority of their lifespan. One major finding was that there was a remarkable decrease in TxNiP protein content in soleus muscle of trained mice compared to nontrained controls. Although a similar decrease (over 50%) in TxNiP levels was observed, the effect of long-term exercise training on TxNiP levels did not quite reach statistical significance in the T.A. muscle. This may be due to the small sample size and variations in the results due to the semiquantitative nature of Western blot techniques. In addition, consistent with the HSP responses, it is also expected that the influence of aerobic exercise on redox regulation would be more prominent in a muscular tissue consisting of mainly slow oxidative muscle fibers. The existing information on the effect of regular exercise on tissue TxNiP levels is, however, scanty. In the only publication in the literature, there was no exercise-mediated effect on the TxNiP level in the rat brain even though there was an increase in the level of TRX-1 protein [37]. Nevertheless, because brain tissue is not actively involved in the metabolic changes during exercise, caution is needed when making a direct comparison of the responses of muscle and brain tissue to exercise. Notably, we detected an increase in the TRX-1/TxNiP ratio in skeletal muscle in response to long-term exercise. Recent reports suggest that an increase in TRX together with a decrease in TxNiP expression could help to prevent various pathologies (reviewed recently by Yoshihara et al. [31]). In addition, we examined the levels of protein carbonyls and 4-HNE adducts, utilized as markers of protein oxidation and lipid peroxidation, respectively, in mice skeletal muscle. After long-term voluntary exercise, the amounts of 4-HNE adducts significantly decreased in T.A. muscle of OE mice, whereas there was no change in the protein carbonyls. These results are consistent with a previous study, which observed a decrease of lipid peroxidation levels after regular exercise training [23, 35]. Based on our findings, we suggest that life-long exercise improves the antioxidant TRX system and TRX-1/TxNiP ratio, which promote resistance to oxidative stress and could be protective against several pathologies related to aging.

A major focus of the current study was to explore ER stress and its association to redox regulation and oxidative stress in response to aging and long-term exercise. Markers of ER stress and UPR, GRP78 and PDI, were not significantly influenced in skeletal muscles by long-term exercise. In the existing literature, several studies have demonstrated that ER stress markers remain unaltered or are even suppressed after moderate regular exercise training, while a single bout of exercise with moderate intensity was reported to induce UPR activation [24, 36, 38]. We observed a minor upregulation of GRP78 and PDI proteins after long-term exercise in T.A. muscle of OE mice. In contrast, the level of these two ER stress-related proteins tended to decrease in soleus muscle of OE mice compared to OS. On the other hand, exercise induced a remarkable attenuation of the ER stress-related apoptosis marker CHOP in soleus muscle of aged mice. The level of CHOP also was observed to be lower in T.A. muscle in response to long-term exercise. These findings are consistent with previous studies, that is, significant reductions were demonstrated in the levels of CHOP mRNA in gastrocnemius muscle of rats after treadmill running exercise [23]. Moreover, in support of the relationship between ER stress and redox regulation disorders, the levels of CHOP and TxNiP protein were strongly correlated in T.A. and soleus muscles of all the studied mice. Although TxNiP has been demonstrated to induce apoptosis through activation of apoptosis-stimulating kinase 1 (ASK1) [39], here we report for the first time, a direct association between TxNiP and the ER stress-related apoptosis marker, CHOP. Our results are also in agreement with a recent study which provided direct evidence of the association of TxNiP with PDI activity and ER stress [40]. In addition, the TRX-1/TxNiP ratio correlated negatively and strongly with the level of CHOP in T.A. and soleus muscles. Therefore, our data demonstrates that lower TxNiP levels can be protective against ER stress-related apoptosis, and furthermore, that exercise training can be useful through decreasing TxNiP levels and improving the TRX-1/TxNiP ratio. We conclude that long-term exercise may lead to ER stress adaptation in skeletal muscle and exert protective effects against future stress and ER stress-related apoptosis.

Furthermore, we examined the effect of long-term exercise on HSP levels in skeletal muscle tissue of mice. As compared to OS mice, we observed an increase in GRP75 protein content (41.1%) in T.A. but not in soleus muscle of OE mice. Moreover, we detected a significant upregulation of stress-inducible HSP70 (118.5%), and constitutively expressed HSC70 (64.8%) proteins in soleus muscle after long-term exercise, while in T.A. muscle, these increases did not reach statistical significance. We postulate that similar to the changes we detected in the redox-regulation markers in response to long-term exercise, the difference in the upregulation of stress-inducible HSPs between soleus and T.A. muscles can be attributed to the difference in their metabolic properties; soleus muscle mostly contains slow oxidative fibers, whereas T.A. mostly contains fast glycolytic fibers [41]. There is a greater recruitment of soleus muscle to moderate aerobic exercise such as voluntary wheel-running. Furthermore, exercise-induced metabolic demands and ROS production are higher in soleus muscle, which ultimately result in the induction of HSPs [42]. Enhanced HSP levels in response to different types of exercise in various skeletal muscles have been well demonstrated. Increases in HSP70 expression after moderate treadmill endurance training in soleus and T.A. muscles of rats were recently reported [42]. The protein levels of HSP72 and HSP25 increased in T.A. muscle of old mice after resistance training [43] and in gastrocnemius muscles of adult mice after voluntary wheel-running [44]. Therefore, in agreement with previous studies, we observed an increase of HSP levels, particularly the constituent HSC70 levels, in response to long-term exercise in old animals. On the other hand, the voluntary exercise training used in our study resulted in a lower extent increase of inducible HSP levels when compared to the situation where more strenuous exercise protocols have been adopted. Moreover, the lower extent of HSP increase after long-term exercise can be associated with a compromised adaptive mechanism of HSP expression in old individuals, as compared to young animals and adults [27].

## 5. Conclusion

Collectively, the current study demonstrated that increased ER stress and ER stress-related apoptosis marker and impairment of the redox regulation system and HSP functions are associated with a sedentary lifestyle of old mice and that these changes occur in a muscle type-specific manner. At the same time, life-long exercise appeared to improve redox regulation and HSP defense, as well as to cause a reduction in the TxNiP level, leading to ER stress adaptation and attenuation of ER stress-related apoptosis in skeletal muscle. These findings provide further evidence that life-long exercise has a protective effect against age-related cellular stress processes.

## Figures and Tables

**Table 1:** Effect of aging and long-term voluntary wheel-running on the levels of 4-hydroxynonenal adducts (4-HNE), lipid hydroperoxides (LPO), protein carbonyls, and heat shock proteins in T.A. and soleus mice muscles.

Levels of 4-HNE adducts, LPO, protein carbonyls, and heat shock proteins				
		Y	OS	OE
T.A.	4-HNE	1.00 ± 0.04	0.88 ± 0.11	0.54 ± 0.013^#^
	LPO	1.00 ± 0.01	1.21 ± 0.01	1.26 ± 0.02
	Protein carbonyls	1.00 ± 0.01	1.02 ± 0.01	1.04 ± 0.01
	HSP25	1.00 ± 0.08	1.14 ± 0.23	1.14 ± 0.10
	HSP60	1.00 ± 0.05	1.05 ± 0.12	1.28 ± 0.11
	HSP90	1.00 ± 0.10	1.38 ± 0.24	1.14 ± 0.07

Soleus	4-HNE	1.00 ± 0.13	0.76 ± 0.18	1.07 ± 0.16
	LPO	1.00	0.94	0.95
	Protein carbonyls	1.00 ± 0.01	1.01 ± 0.01	1.01 ± 0.01
	HSP25	1.00 ± 0.06	1.08 ± 0.54	1.55 ± 0.19
	HSP60	1.00 ± 0.07	0.71 ± 0.07	0.72 ± 0.05
	HSP90	1.00 ± 0.07	0.89 ± 0.42	1.19 ± 0.13

Data are mean ± SEM. Groups are as follows: Y, young 3-month-old mice; OS, sedentary 24-month-old mice; OE, 24-month-old mice after 21 months of voluntary wheel-running. *n* = 12 for Y, *n* = 5 for OS, and *n* = 5 for OE. Difference due to exercise: ^#^*P* < 0.05.

**Table 2:** Correlations between ER stress and redox regulation markers in the T.A. and soleus muscles of mice.

ER stress	Redox regulation	Correlation coefficient	*p* value
		T.A./soleus muscle	T.A./soleus muscle
GRP78	TRX-1	0.479/0.728	0.018/0.001
PDI	TRX-1	0.505/0.789	0.012/0.001
CHOP	TxNiP	0.791/0.629	0.001/0.002
CHOP	TRX-1-TxNiP	−0.579/−0.687	0.004/0.001

Correlation analysis was performed by Spearman's test. Correlation data was obtained from all studied mice (*n* = 22). Significance level was set at *p* < 0.05.
